# Primary Reasons for Osteopathic Consultation: A Prospective Survey in Quebec

**DOI:** 10.1371/journal.pone.0106259

**Published:** 2014-09-03

**Authors:** Chantal Morin, Andrée Aubin

**Affiliations:** 1 Centre ostéopathique du Québec, Montréal, Québec, Canada; 2 School of Rehabilitation, Faculty of Medicine and Health Sciences, University of Sherbrooke, Sherbrooke, Québec, Canada; Iran University of Medical Sciences, Iran, Islamic Republic of

## Abstract

**Background:**

Osteopathy is an increasingly popular health care modality to address pain and function in the musculoskeletal system, organs and the head region, as well as functional somatic syndromes. Although osteopathy is recommended principally in guidelines for management of back pain, osteopaths' scope of practice is wide, albeit poorly defined. In order to understand better the practice of osteopathy, this study aimed to investigate the most common reasons for osteopathic consultations in clinical settings in Quebec.

**Methods:**

A prospective survey of members of the Registre des ostéopathes du Québec was conducted to examine demographics in osteopathic practices, as well as patients' primary reasons for consultations over a two-week period. The questionnaire was devised following a literature review and refined and verified with two stages of expert input.

**Results:**

277 osteopaths (60.1% response rate) responded to the survey notice. 14,002 patients' primary reasons for consultations were reported in completed questionnaires and returned by practicing osteopaths. Musculoskeletal pain located in the spine, thorax, pelvis and limbs was the most common reason for consultations (61.9%), with females consulting most commonly for cervical pain and males for lumbar pain. Perinatal and paediatric (11.8%), head (9.1%), visceral (5.0%) and general concerns (4.8%) were the other most common reasons for consultations. Preventive care represented the remaining 0.3%.

**Interpretation:**

The nature of primary reasons for osteopathic consultations, coupled with documented satisfaction of patients with this approach, suggest a future for multidisciplinary collaborative health care including osteopathy. Results of this survey may contribute to informing physicians and others pending regulation of Quebec osteopaths, and also provide direction for future clinical research and guidelines development.

## Introduction

Osteopathy is based on manual contact for medical assessment and treatment. According to Benchmarks for training in osteopathy guidelines published by the World Health Organization (WHO), the osteopathic practitioners use a wide variety of therapeutic manual techniques to improve physiological function and/or support homeostasis that has been altered by somatic (body framework) dysfunction. Examples include impaired or altered function of components of the somatic system; skeletal, arthrodial and myofascial structures; and vascular, lymphatic, and neural elements [1]. Osteopathy is based on the principle that the structure and functions of the body are closely integrated, and that a person's wellbeing requires the neurological, musculoskeletal, circulatory and visceral structures to work in balance together. Osteopathic practice thus aims to restore (and maintain) a person's body to its overall natural state of wellbeing [2]. Osteopathy is an increasingly popular approach for somatic dysfunctions [1]. Disordered physiology may manifest as functional somatic syndromes, characterised by patterns of persistent bodily complaints that may occur independently from pathological changes [3].

Canadians' use of osteopathy doubled between 1997 and 2006; much of this occurred in Quebec, where utilization increased from 3% to 11% during this period [4]. Osteopathy is also the complementary therapy that is the most frequently recommended by general practitioners in Quebec [5].

In light of increasing use of osteopathy by Quebecers, including as a front line services, regulation seems desirable, and indeed the Office des professions du Québec intends to regulate the osteopathic profession in the province [6], to protect the osteopath's title, define reserved acts and shift education from private schools to a university Master's degree. Non-physician osteopaths are already regulated in many countries (United Kingdom (UK), Australia, Finland, New Zealand, France and Switzerland) [7]. Osteopathy is recommended mainly in guidelines for management of back pain [8], [9], [10], [11], [12], but the scope of practice of osteopaths is wide, albeit poorly defined. Only a few studies in countries where osteopathy is a regulated profession reported results of surveys about profiles of practice including primary reasons for osteopathic consultations [13], [14], [15], [16], [17]. These recent surveys are one day snap shots documenting many aspects of osteopathic practice at the same time. They have limited rates of participation (3.4% to 38.9%), and report on small numbers of patients for each osteopath participant. The total number of patients reporting a primary reason for consultation is limited (799 to 2238 patients). To our knowledge, no published study has reported the most common reasons for osteopathic consultation for all patients seen over more than one day, for more than few patients per osteopath, or in relation to gender. As well, no study has been conducted specifically in Quebec, or in Canada. With the prospect of regulation and the increasing popularity of osteopathy, there is a need to better understand its practice in Quebec, in particular the most common reasons for consultation.

Although effectiveness of osteopathy for various conditions needs to be further documented, the aim of this study was to investigate the most common reasons for osteopathic consultations in clinical settings in Quebec for all patients seen during a two-week period. This knowledge is needed to inform not only regulatory bodies, but also health care providers in order to promote patient centered care including open physician-patient communication related to osteopathic consultations, and to establish research priorities.

## Methods

### Setting and participants

All members of the Registre des Osteopathes du Québec (ROQ) practicing in the province were eligible to participate. The ROQ (now known as Ostéopathie Québec) was the largest professional body of osteopaths in Quebec in 2011–2012, representing the majority of provincial osteopaths with training according to the WHO Benchmarks for training in osteopathy [1]. The Human Research and Ethics Committee of the Centre Hospitalier Universitaire de Sherbrooke provided approval for the study. Precise written instructions provide along with the questionnaire included reassurance that information provided was only to examine the most common reasons for osteopathic consultation in Quebec, and that all data would be treated and reported anonymously. The only patient data collected were gender, age category (children from birth to 14 years, or adult 15 years and older) and the leading reason for consulting in osteopathy.

### Study design

For this descriptive study, a prospective self-administrated survey was devised based upon a literature review, and adapted for the Quebec provincial context. The questionnaire captured demographic information about osteopaths, limited patient data (age category, gender) and the primary reason for consultation for each patient. The use of anatomical sites of symptoms as categories for adult patients and concerns for paediatric patients is consistent with the standardised data collection in Part two (presenting symptoms) of the tool developed by the National Council for Osteopathic Research (NCOR) in UK [14], [17], [18]. Although the NCOR data collection tool was regarded as being useful for a cross-sectional survey, clinicians expressed the view that the whole tool was too long to use on a daily basis in practice [18]. The current study was designed to optimize the participation rate with a short questionnaire to be completed on a day-to day basis in clinic, to capture the most common reasons for consultation over a two-week period, with a sample allowing comparisons between genders.

Questionnaires were completed during a consecutive two-week working period at the convenience of each osteopath, between October 2011 and March 2012. The survey was announced in a communication by the ROQ, and the questionnaire was distributed to all osteopathic practitioners registered with the ROQ. The first postal mailing occurred in October 2011 (n = 454), followed by an email reminder two weeks later. The questionnaire was attached to an email reminder in December 2011 and again in February 2012. The completed survey was returned either by postal mail or by email to the principal investigator. In addition to the primary reason for consultation of each patient, osteopaths had to indicate the total number of females, males and children seen in the two-week period of the survey. A research assistant verified the equivalence of the total numbers indicated, and the data provided regarding individual patients.

### Primary measurements

The questionnaire sought demographic information about the osteopath, years of experience, time spent with patients for a single consultation, practice profiles (type of practice), percentage of the practice that entails an exclusive osteopathic approach, and each patient's primary reason for the consultation. Primary reasons of consultation were categorized according to the anatomical site of the symptom (pain or dysfunction) except for perinatal and paediatric which were categorized according to the concern. Categories are: 1) Upper limb; 2) Lower limb; 3) Spine or pelvis; 4) Visceral; 5) Head; 6) Perinatal and paediatric concerns; 7) General concerns; or 8) Any other reasons (Questionnaire in French, Appendix S1). These eight categories included finer sub-categories, differentiated according to patient gender. This straightforward classification scheme avoided relying on a variety of practitioners' categories, on the results of physical assessment, or on the presumed origin of the symptoms [19]. Osteopathic practitioners could add items as needed.

A preliminary version of the questionnaire included sociodemographic information, and areas of symptoms relevant defined in other similar surveys including the standardised data collection tool developed by the NCOR [18]. Two experts, each with more than 10 years of clinical experience in osteopathy, one of whom also had a research background, established face validity of the initial version of the questionnaire. Each expert also provided feedback and participated in discussions to improve the questionnaire. It was further refined in accordance with clarifications recommended by four osteopaths, who pre-tested the instrument for a typical week of work.

### Statistical analysis

Descriptive statistics (frequencies and percentages) were used to analyse demographics of respondents and to explore data on common reasons for consultations. Demographics (gender and years of experience) of respondents were compared to those of non-respondent members of the ROQ using chi-square and T-tests. Chi-square tests were further used to compare reasons for consulting between patients' gender in the entire sample (regardless of their osteopath). Statistical significance was set at *p*<0.001 after the Bonferroni correction was applied for multiple comparisons. Analyses were conducted using SPSS (version 17.0, SPSS Inc, Chicago, IL, USA).

## Results

Of the 454 osteopaths initially mailed, 274 (60.1%) responded to the survey notice, of whom 245 were currently working and completed the prospective two week questionnaire. Four questionnaires were completed incorrectly and rejected in the analysis (multiple primary reasons for consulting were given per patient (n = 3); or data collection was only a one week period rather than a 2 week working period (n = 1)). Gender (p = 0.97) and years of experience (p = 0.17) were similar between osteopath respondents and non-respondents (data not shown). Characteristics of osteopath respondents are shown in Table 1. Osteopaths spent a mean of 55 minutes (range 30 to 75 minutes standard deviation (SD) = 8.79) with each patient. On average, they devoted 92.9% of their practice specifically to osteopathy. The total number of females, males and children treated indicated by each osteopath, and the data provided regarding individual patients were consistent in all questionnaires included in the analysis.

**Table 1:** Characteristics of osteopath respondents (N = 241).

	Frequency	(%)
**Gender**		
Female	160	(66.4)
**Experience**		
0–5 years	69	(28.6)
5–10 years	54	(22.4)
10–15 years	50	(20.7)
15 and more years	68	(28.2)
**Areas of practice** *		
General	237	(98.3)
Perinatal	106	(44.0)
Sports	68	(28.2)
Urogynecology	48	(20.0)

* An osteopath may cover more than one area of practice.

**Figure 1:**
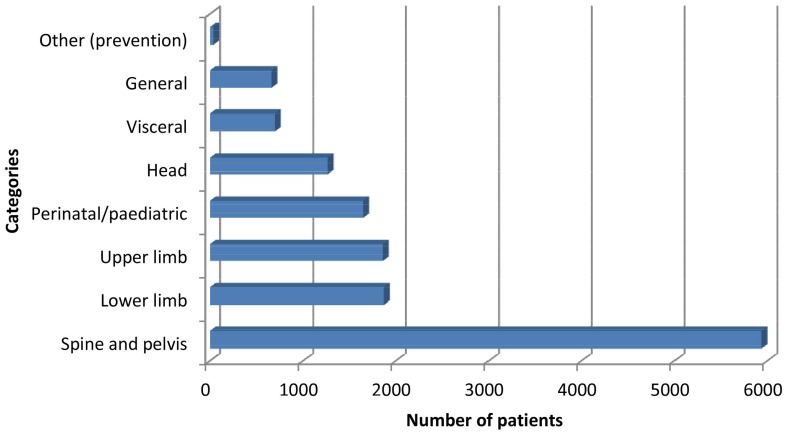
Frequencies of primary reason of consultation for all patients (n = 14002).

Overall, respondents treated a total of 14002 patients during the two-week working period, including 8739 females (62.4%), 3826 males (27.3%) and 1437 children (10.3%). A very high percentage (98.3%) of osteopaths had a general type of practice and nearly half treat children. Spinal, thorax and pelvic symptoms (42.4%) were the predominant primary reasons for osteopathic consultation, followed by lower limb (13.4%) and upper limb (13.3%) symptoms (Figure 1). Thus, musculoskeletal symptoms located in the spine, pelvis, thorax or limbs represented 69.1% of the primary reasons to consult an osteopath. Although those musculoskeletal symptoms were the leading reasons for both males and females, some differences were observed between genders (Table 2), with females consulting significantly more frequently for cervical and hip symptoms than males, while males consulted more frequently for lumbar, as well as shoulder and elbow symptoms.

**Table 2:** Reasons for osteopathic consultation related to pain and dysfunction in spine, pelvis and limbs.

Location	All* (N = 14002)	Female (n = 8739)	Male (n = 3826)	
	Frequency (%; 95%CI)	Frequency (%; 95%CI)	Frequency (%; 95%CI)	*P* value†
**Spine and pelvis**	**5940 (42.4; 41.6–43.2)**	**3997 (45.7; 44.7–46.8)**	**1943 (50.8; 49.2–52.4)**	
Cervical	1796 (12.8; 12.3–13.4)	1321 (15.1; 14.4–15.9)	475 (12.4; 11.4–13.5)	<0.001
Dorsal or thorax	993 (7.1; 6.7–7.5)	656 (7.5; 7.0–8.1)	337 (8.8; 7.9–9.8)	0.013
Lumbar	2025 (14.5; 13.9–15.1)	1202 (13.8; 13.0–14.5)	823 (21.5; 20.2–22.8)	<0.001
Pelvis	662 (4.7; 4.4–5.1)	484 (5,5; 5.1–6.0)	178 (4.7; 4.0–5.4)	0.041
Spine, general	439 (3.1; 2.9–3.4)	316 (3.6; 3.2–4.0)	123 (3.2; 2.7–3.8)	0.260
Hernia or scoliosis	25 (0.2; 0.1–0.3)	18 (0.2; 0.1–0.3)	7 (0.2; 0.09–0.4)	0.790
**Lower limb**	**1873 (13.4; 12.8–14.0)**	**1262 (14.4; 13.7–15.2)**	**611 (16.0; 14.8–17.2)**	
Hip	452 (3.2; 3.0–3.5)	352 (4.0; 3.6–4.5)	100 (2.6; 2.2–3.2)	<0.001
Knee	489 (3.5; 3.2–3.8)	300 (3.4; 3.1–3.8)	189 (4.9; 4.3–5.7)	<0.001
Ankle and foot	441 (3.2; 2.9–3.5)	278 (3.2; 2.8–3.6)	163 (4.3; 3.7–5.0)	0.002
Lower limb, general	141 (1.0; 0.9–1.1)	99 (1.1; 0.9–1.4)	42 (1.1; 0.8–1.5)	0.864
Referred pain	350 (2.5; 2.3–2.7)	233 (2.7; 2.4–3.0)	117 (3.1; 2.6–3.7)	0.219
**Upper limb**	**1863 (13.3; 12.8–13.9)**	**1198 (13.7; 13.0–14.4)**	**665 (17.4; 16.2–18.6)**	
Shoulder	1108 (7.9; 7.5–8.3)	696 (8.0; 7.4–8.6)	412 (10.8; 9.8–11.8)	<0.001
Elbow	199 (1.4; 1.2–1.6)	106 (1.2; 1.0–1.5)	93 (2.4; 2.0–3.0)	<0.001
Wrist and hand	202 (1.4; 1.2–1.6)	145 (1.7; 1.4–2.0)	57 (1.5; 1.1–1.9)	0.487
Upper limb, general	147 (1.1; 0.9–1.2)	105 (1.2; 1.0–1.4)	42 (1.1; 0.8–1.5)	0.619
Referred pain	207 (1.5; 1.3–1.7)	146 (1.7; 1.4–2.0)	61 (1.6; 1.2–2.0)	0.757

95%CI: 95% confidence interval.

* All includes adults and children.

† *P*-value for differences between frequency of consultation according to patient gender. Statistically significant difference between genders' was defined as *p*<0.001 using the Bonferroni correction due to multiple comparisons.

**Figure 2:**
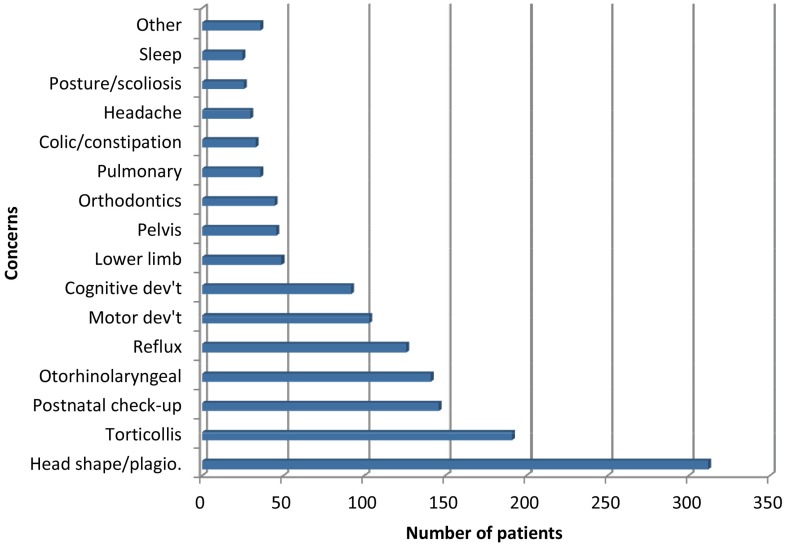
Frequencies of primary reason for paediatric consultations (Children <15 y, n = 1437).

Perinatal and paediatric concerns combined (11.8%), including consultations both for pain or discomfort experienced by pregnant women (n = 211) and for children below 15 years of age (n = 1437), was the second most common category of reasons for osteopathic consultations, after musculoskeletal complaints located in the spine, thorax, pelvis or limbs. Paediatric consultations were for a wide range of concerns; proportions of each of these categories are presented in Figure 2.

Symptoms localized in the head region (9.1%) were the third most common reason for osteopathic consultations, followed by visceral (5.0%), general concerns (4.7%) and preventive care (0.3%) (Table 3). For those categories, females consulted significantly more frequently for migraine, headache, urogenycological concerns and chronic pain associated with systemic dysfunction (e.g. fibromyalgia) than did males. Urogenycological concerns refer to urinary problems such as incontinence and perineal re-education for both females and males. In addition, some female patients also consulted for reproductive system dysfunction.

**Table 3:** Reasons for osteopathic consultation related to visceral region, head region, general concerns, preventive care, and perinatal and paediatric concerns.

Location or concern	All* (N = 14002)	Female (n = 8739)	Male (n = 3826)	
	Frequency (%; 95%CI)	Frequency (%; 95%CI)	Frequency (%; 95%CI)	*P* value†
**Visceral**	**703 (5.0; 4.7–5.4)**	**549 (6.3; 5.8–6.8)**	**154 (4.0; 3.4–4.7)**	
Digestive	371 (2.7; 2.4–2.9)	276 (3.2; 2.8–3.6)	95 (2.5; 2.0–3.0)	0.040
Pulmonary	96 (0.7; 0.6–0.8)	67 (0.8; 0.6–1.0)	29 (0.8; 0.5–1.1)	0.959
Urogynecology	199 (1.4; 1.2–1.6)	180 (2.1; 1.8–2.4)	19 (0.5; 0.3–0.8)	<0.001
Other visceral concerns‡	37 (0.3; 0.2–0.4)	26 (0.3; 0.2–0.4)	11 (0.3; 0.2–0.5)	0.924
**Head**	**1269 (9.1; 8.6–9.6)**	**972 (11.1; 10.5–11.8)**	**297 (7.8; 7.0–8.6)**	
Migraine	235 (1.7; 1.5–1.9)	194 (2.2; 1.9–2.5)	41 (1.1; 0.8–1.4)	<0.001
Headache	377 (2.7; 2.4–3.0)	297 (3.4; 3.0–3.8)	80 (2.1; 1.7–2.6)	<0.001
Dizziness, vertigo	153 (1.1; 0.9–1.3)	110 (1.3; 1.1–1.5)	43 (1.1; 0.8–1.5)	0.526
Facial pain	72 (0.5; 0.4–0.6)	53 (0.6, 0.5–0.8)	19 (0.5; 0.3–0.8)	0.453
Otorhinolaryngeal	161 (1.2; 1.0–1.4)	114 (1.3; 1.1–1.6)	47 (1.2; 0.9–1.6)	0.727
Orthodontics	34 (0.2; 0.1–0.3)	26 (0.3; 0.2–0.4)	8 (0.2; 0.1–0.4)	0.380
Temporomandibular joint	195 (1.4; 1.2–1.6)	151 (1.7; 1.5–2.0)	44 (1.2; 0.9–1.5)	0.016
Commotion or eye-related	42 (0.3; 0.2–0.4)	27 (0.3; 0.2–0.4)	15 (0.4; 0.2–0.6)	0.458
**General concerns**	666 (4.8; 4.4–5.1)	526 (6.0; 5.5–6.5)		
Fatigue	233 (1.7; 1.5–1.9)	184 (2.1; 1.8–2.4)	49 (1.3; 1.0–1.7)	0.002
Mood	58 (0.4; 0.3–0.5)	42 (0.5; 0.4–0.6)	16 (0.4; 0.3–0.7)	0.635
Sleep	85 (0.6; 0.5–0.7)	59 (0.7; 0.5–0.9)	26 (0.7; 0.5–1.0)	0.978
Chronic pain	226 (1.6; 1.4–1.8)	189 (2.2; 1.9–2.5)	37 (1.0; 0.7–1.3)	<0.001
Other general concerns§	64 (0.5; 0.4–0.6)	52 (0.6; 0.5–0.8)	12 (0.3; 0.2–0.5)	0.041
**Preventive care**	40 (0.3; 0.2–0.4)	24 (0.3; 0.2–0.4)	16 (0.4; 0.2–0.7)	0.189
**Perinatal and paediatric concerns**	1648 (11.8; 11.3–12.3)	211 (2.4; 2.1–2.7)		

95%CI: 95% confidence interval.

* All includes men, women and children.

† *P*-value for differences in frequency of reason for consultation according to patient gender. Statistically significant difference between genders' was defined as *p*<0.001 using the Bonferroni correction due to multiple comparisons.

‡ Abdominal pain, post-surgical adhesions, skin problems.

§ Hormonal balance, degenerative diseases, homeostasis and vitality, circulatory problems and depression.

If a significance level of 5% is used instead of the 0.1% threshold, females also consult more than males for fatigue, temporomandibular disorders, pelvic pain, digestive problems and other general questions whereas males consult more than females for pain in ankles or feet and back or chest. The dataset is available upon request.

## Interpretation

This first survey of osteopaths in Quebec reports on 241 practitioners and 14002 patients, seen over a two-week period. The high initial response rate (60.1% of the ROQ membership), with no significant differences in gender and years of experience between respondents and non-respondents, indicate that findings are representative of the most common reasons for consultation among osteopath members of the ROQ, the largest professional body of osteopaths in Quebec in 2011–2012.

Osteopaths were predominately consulted for musculoskeletal pain and dysfunction located in the spinal, pelvis, thorax and limbs. Lumbar (particularly in males) and cervical (particularly in females) regions were the most frequent pain locations, followed by shoulder, dorsal and costal regions. Perinatal and paediatric concerns were also frequent; principally for head shape, as well as torticollis. Patients also consulted for a variety of other concerns in the head region as well as for visceral, general and prevention purposes.

The finding that musculoskeletal pain and dysfunction in the spine, pelvis, thorax, and limbs was the primary reason for osteopathic consultation is consistent with recent surveys on osteopathic practices in general populations, across the UK, France and Australia [13], [14], [15], [16], [17]. None of previous surveys compared male's and female's reasons for consultation in osteopathy.

The trends observed for osteopathic consultation in the present study mirror known patterns of symptoms and physician consultation. In Quebec, males are more likely than females to consult a physician repeatedly for low back pain [20], while cervical pain was more frequently documented in females than in males [21]. A higher prevalence of headache [22], [23] and chronic pain [24] have also been observed in Canadian females than males.

Paediatric osteopathic consultations ranged from 8% to 12% across previous surveys [13], [14], [15] but few details are available on primary reasons for consultation. An American study using administrative data on clinical diagnosis of paediatric patients [25] yielded similar observations, with consultations predominantly for torticollis and skull/face deformity, otitis media, infant feeding problems, muscle spasms and gastrointestinal concerns.

International recommendations for early management of musculoskeletal symptoms [26] and potentially severe impacts of spinal problems on health status [27] necessitate consideration of options for complementary care. Multidisciplinary care including professionals such as osteopaths, whose clinical interests and expertise focus on specific treatments for musculoskeletal and functional problems, must be pursued, along with ongoing research to document efficacy and cost-effectiveness of interventions [26], [28]. Knowledge of common reasons for osteopathic consultation may facilitate open physician-patient communication about best approaches for musculoskeletal and functional problems in multidisciplinary care. While patients express satisfaction with osteopathic treatment results, explanations given by osteopaths and overall health outcomes [14], [29], physicians should be able to help patients to make the best choices in health care, including consideration of complementary therapies like osteopathy [30], [31], [32], [33].

### Limitations and strengths

The chief limitation of the questionnaire design was that only the primary reason for each consultation was collected. We were concerned that documentation of multiple reasons, requiring more time for questionnaire completion by osteopaths, might have reduced the participation rate.

Patients are seldom seen twice in the same month in osteopathic care, so the questionnaire did not capture if a patient was treated more than once during the two-week working period. Thus, a small amount of duplication may have occurred. Although surveys were verified for accuracy, it is possible that osteopaths may have omitted some patients during the two-week working period. This is a possible source of bias, although the scale is unknown alongside the 14002 documented reasons for consultation. Finally, given the number of statistical tests carried out, it is possible that type I errors occurred; however, the Bonferroni correction was applied to minimize this likelihood.

The main strengths of this study are the prospective design to reduce inaccuracies with recall, a high response rate, and inclusion of patient gender. The results of our survey provide the first general clinical overview of patients' reasons to consult an osteopath member of the ROQ.

### Conclusion and future direction

Osteopaths treat both adults and children, mainly for musculoskeletal pain and dysfunction located in the spine, pelvis, thorax and limbs, but also for head region complaints, visceral and general concerns, as well as prevention, for both adults and children. The nature of primary reasons for osteopathic consultations, coupled with documented satisfaction of patients with this approach, suggest a future for multidisciplinary collaborative health care including osteopathy. With regulation of osteopathic practice pending in Quebec, results of this survey could contribute to informing regulators, to developing clinical guidelines and establishing clinical research priorities. Further research is needed to investigate safety and effectiveness of osteopathic practice for a variety of conditions, including patients' outcomes and satisfaction. Studies of effective interprofessional collaboration between physicians and osteopaths will facilitate safe and efficient patient centered care.

## Supporting Information

Appendix S1 **Questionnaire in French.**
(PDF)
