# Correction: Primary Reasons for Osteopathic Consultation: A Prospective Survey in Quebec

**DOI:** 10.1371/journal.pone.0121180

**Published:** 2015-03-20

**Authors:** The PLOS ONE Staff

There is an error in the first sentence of the “Results” subsection of the Abstract. The correct sentence is: 274 osteopaths (60% response rate) responded to the survey notice.
