# Supplementary material for: Primary Reasons for Osteopathic Consultation: A Prospective Survey in Quebec
Source: PLoS One. 2014 Sep 3;9(9):e106259. doi: 10.1371/journal.pone.0106259 (PMC4153609; doi:10.1371/journal.pone.0106259)
Supplement: Appendix S1 — Questionnaire in French. (PDF) [file pone.0106259.s001.pdf]

SONDAGE SUR LES MOTIFS DE CONSULTATION

**Région de pratique :**					**Genre :** F ❐ H ❐
**Type de pratique :**	Général ❐	Périnatalité ❐	Sportif ❐	Uro-génital ❐	
**Exp. comme ostéo diplômé(e) :**	0-3 ans ❐	3-5 ans ❐	5-10 ans ❐	10-15 ans ❐	15 ans + ❐
**Temps par séance :**			**% de pratique ostéopathique :**		
**Nombre total de clients pendant le sondage :**		**# H :**	**# F :**	**# enfants (0-14 ans) :**	

**PROBLÈMES MUSCULO-SQUELETTIQUES**											
**Douleurs du membre supérieur**									**Si 10+, indiquez le # exact ⇩ :**		
Épaule	**F**	1	2	3	4	5	6	7	8	9	10	
	**H**	1	2	3	4	5	6	7	8	9	10	
Coude	**F**	1	2	3	4	5	6	7	8	9	10	
	**H**	1	2	3	4	5	6	7	8	9	10	
Poignet et main	**F**	1	2	3	4	5	6	7	8	9	10	
	**H**	1	2	3	4	5	6	7	8	9	10	
Tout le membre supérieur	**F**	1	2	3	4	5	6	7	8	9	10	
	**H**	1	2	3	4	5	6	7	8	9	10	
Douleurs référées au membre supérieur	**F**	1	2	3	4	5	6	7	8	9	10	
	**H**	1	2	3	4	5	6	7	8	9	10	
**Douleurs du membre inférieur**									**Si 10+, indiquez le # exact ⇩ :**		
Hanche	**F**	1	2	3	4	5	6	7	8	9	10	
	**H**	1	2	3	4	5	6	7	8	9	10	
Genou	**F**	1	2	3	4	5	6	7	8	9	10	
	**H**	1	2	3	4	5	6	7	8	9	10	
Cheville et pied	**F**	1	2	3	4	5	6	7	8	9	10	
	**H**	1	2	3	4	5	6	7	8	9	10	
Tout le membre inférieur	**F**	1	2	3	4	5	6	7	8	9	10	
	**H**	1	2	3	4	5	6	7	8	9	10	
Douleurs référées au membre inférieur	**F**	1	2	3	4	5	6	7	8	9	10	
	**H**	1	2	3	4	5	6	7	8	9	10	
**Douleurs vertébrales et du bassin**									**Si 10+, indiquez le # exact ⇩ :**		
Douleurs cervicales	**F**	1	2	3	4	5	6	7	8	9	10	
	**H**	1	2	3	4	5	6	7	8	9	10	
Douleurs dorsales et costales	**F**	1	2	3	4	5	6	7	8	9	10	
	**H**	1	2	3	4	5	6	7	8	9	10	
Douleurs lombaires et lombo-sacrées	**F**	1	2	3	4	5	6	7	8	9	10	
	**H**	1	2	3	4	5	6	7	8	9	10	
Douleur bassin (sacro-iliaque et coccyx)	**F**	1	2	3	4	5	6	7	8	9	10	
	**H**	1	2	3	4	5	6	7	8	9	10	
Douleurs vertébrales étendues (non-spécifiques)	**F**	1	2	3	4	5	6	7	8	9	10	
	**H**	1	2	3	4	5	6	7	8	9	10	

Autres problèmes musculo-squlettiques (indiquez le motif de consultation et le genre du sujet)

F ❐<br>H ❐	F ❐<br>H ❐	F ❐<br>H ❐

**DOULEURS OU DYSFONCTIONNEMENTS VISCÉRAUX**									**Si 10+, indiquez le # exact ⇩ :**		
Troubles digestifs	**F**	1	2	3	4	5	6	7	8	9	10	
	**H**	1	2	3	4	5	6	7	8	9	10	
Troubles pulmonaires et de la respiration	**F**	1	2	3	4	5	6	7	8	9	10	
	**H**	1	2	3	4	5	6	7	8	9	10	
Troubles gynécologiques	**F**	1	2	3	4	5	6	7	8	9	10	
	**H**	1	2	3	4	5	6	7	8	9	10	

Autres douleurs ou dysfonctionnements viscéraux (indiquez le motif de consultation et le genre du sujet)

F ❐<br>H ❐	F ❐<br>H ❐	F ❐<br>H ❐

DOULEURS OU DYSFONCTIONNEMENTS DE LA TÊTE												Si 10+, indiquez le # exact ⇩ :
Migraines	**F**	1	2	3	4	5	6	7	8	9	10	
	**H**	1	2	3	4	5	6	7	8	9	10	
Céphalées/maux de tête	**F**	1	2	3	4	5	6	7	8	9	10	
	**H**	1	2	3	4	5	6	7	8	9	10	
Étourdissements et/ou vertiges	**F**	1	2	3	4	5	6	7	8	9	10	
	**H**	1	2	3	4	5	6	7	8	9	10	
Douleurs du visage	**F**	1	2	3	4	5	6	7	8	9	10	
	**H**	1	2	3	4	5	6	7	8	9	10	
Troubles de la sphère ORL adulte	**F**	1	2	3	4	5	6	7	8	9	10	
	**H**	1	2	3	4	5	6	7	8	9	10	
Accompagnement à l’orthodontie	**F**	1	2	3	4	5	6	7	8	9	10	
	**H**	1	2	3	4	5	6	7	8	9	10	
Problèmes de l’ATM	**F**	1	2	3	4	5	6	7	8	9	10	
	**H**	1	2	3	4	5	6	7	8	9	10	

Autres douleurs ou dysfonctionnements de la tête (indiquez le motif de consultation et le genre du sujet)

F ❐<br>H ❐	F ❐<br>H ❐	F ❐<br>H ❐

PÉRINATALITÉ											
**Femmes enceintes**											**Si 10+, indiquez le # exact ⇩ :**
Suivi de grossesse (tous motifs confondus)	1	2	3	4	5	6	7	8	9	10	
**Bébés et enfants (0 à 14 ans)**											**Si 10+, indiquez le # exact ⇩ :**
Contrôle suite à la naissance	1	2	3	4	5	6	7	8	9	10	
Torticolis	1	2	3	4	5	6	7	8	9	10	
Déformations du crâne (ex. plagiocéphalie)	1	2	3	4	5	6	7	8	9	10	
Otites/sinusite/sphère ORL	1	2	3	4	5	6	7	8	9	10	
Reflux et régurgitations	1	2	3	4	5	6	7	8	9	10	
Troubles du dév. moteur	1	2	3	4	5	6	7	8	9	10	
Trouble du dév. psycho-cognitif	1	2	3	4	5	6	7	8	9	10	
Troubles pulmonaires/respiration	1	2	3	4	5	6	7	8	9	10	

Autres motifs concernant les bébés et les enfants (indiquez le motif de consultation)

TROUBLES GÉNÉRAUX												Si 10+, indiquez le # exact ⇩ :
Fatigue	**F**	1	2	3	4	5	6	7	8	9	10	
	**H**	1	2	3	4	5	6	7	8	9	10	
Humeur	**F**	1	2	3	4	5	6	7	8	9	10	
	**H**	1	2	3	4	5	6	7	8	9	10	
Sommeil	**F**	1	2	3	4	5	6	7	8	9	10	
	**H**	1	2	3	4	5	6	7	8	9	10	
Douleurs chroniques et systémiques	**F**	1	2	3	4	5	6	7	8	9	10	
	**H**	1	2	3	4	5	6	7	8	9	10	

Autres troubles généraux (indiquez le motif de consultation et le genre du sujet)

F ❐<br>H ❐	F ❐<br>H ❐	F ❐<br>H ❐

AUTRES MOTIFS DE CONSULTATION NON ENCORE CITÉS (indiquez le motif de consultation et le genre du sujet)

F ❐<br>H ❐	F ❐<br>H ❐	F ❐<br>H ❐
F ❐<br>H ❐	F ❐<br>H ❐	F ❐<br>H ❐
